# IgG3-antigen complexes are deposited on follicular dendritic cells in the presence of C1q and C3

**DOI:** 10.1038/s41598-017-05704-3

**Published:** 2017-07-14

**Authors:** Lu Zhang, Zhoujie Ding, Birgitta Heyman

**Affiliations:** Department of Medical Biochemistry and Microbiology, Uppsala University, Box 582, BMC, SE-751 23 Uppsala, Sweden

## Abstract

IgG3, passively administered together with small proteins, induces enhanced primary humoral responses against these proteins. We previously found that, within 2 h of immunization, marginal zone (MZ) B cells capture IgG3-antigen complexes and transport them into splenic follicles and that this requires the presence of complement receptors 1 and 2. We have here investigated the localization of IgG3 anti-2, 4, 6-trinitrophenyl (TNP)/biotin-ovalbumin-TNP immune complexes in the follicles and the involvement of classical versus total complement activation in this process. The majority (50–90%) of antigen inside the follicles of mice immunized with IgG3-antigen complexes co-localized with the follicular dendritic cell (FDC) network. Capture of antigen by MZ B cells as well as antigen deposition on FDC was severely impaired in mice lacking C1q or C3, and lack of either C1q or C3 also impaired the ability of IgG3 to enhance antibody responses. Finally, IgG3 efficiently primed for a memory response against small proteins as well as against the large protein keyhole limpet hemocyanine.

## Introduction

Complement is generally known as part of our innate immune response, particularly efficient in causing osmotic lysis of pathogens. However, complement is also important for the generation of antibody responses against thymus-dependent as well as thymus-independent antigens. Animals and humans lacking complement factors C1q, C2, C4, C3, or complement receptors 1 and 2 (CR1/2), have severely impaired antibody responses (reviewed in refs 1 and 2). It is generally assumed that the role of these different factors is mediated through CR1/2 (i) because mice lacking these receptors have a similar phenotype as mice lacking C1q, C2, C4, or C3, and (ii) because the ligands for CR1/2 are subfragments of C3 (iC3b, C3dg, and C3b for CR1; iC3b and C3dg for CR2) generated with the help of C1q, C2, and C4. In mice, CR1/2 are alternative splice forms of the Cr2 gene and Cr2 knock-out (KO) mice therefore lack both receptors. However, recently a mouse strain selectively lacking the longer splice form, CR1, was generated by deleting only the CR1-specific exons from the Cr2 gene^3^. CR1/2 are expressed on B cells and follicular dendritic cells (FDC) and, using the selective CR1 KO strain, it was shown that FDC preferentially express CR1 and B cells preferentially CR2^3^. Several molecular mechanisms explaining how CR1/2 can enhance an antibody response have been discussed. Co-crosslinking of BCR and the CD19/CR2 co-receptor complex on the B cell surface lowers the threshold for B cell activation *in vitro* ^4, 5^ and antigen-complement complexes may serve as cross-linkers in the *in vivo* situation. Marginal zone (MZ) B cells express high levels of CR1/2, shuttle between the MZ and the splenic B cell zone (follicle) and transport antigen-complement complexes into the follicle in which they are delivered to CR1/2^+^ FDC^6–8^. Thus, B cell signaling, MZ B cell-mediated transport, and/or capture and presentation by FDC may explain the involvement of CR1/2 in antibody responses.

All three pathways of complement activation lead to cleavage of factor C3 and thereby to the generation of ligands for CR1/2. However, while lack of C1qA, and as a consequence lack of the entire C1q molecule, severely impairs antibody responses^9, 10^, lack of factor B of the alternative pathway^11^ or mannose-binding lectin of the lectin pathway^12, 13^ does not have a severe impact on antibody responses. The crucial role for the classical pathway suggests that antibodies, considered to be the most efficient classical pathway activators, play an important role. IgM and IgG3 are two isotypes that have the capacity to upregulate antibody responses via complement. This is an example of antibody feedback regulation where antibodies, either passively administered or endogenously produced, form immune complexes with their specific antigens and influence the active antibody responses against the antigens. Depending on the antibody classes and the types of antigen, complete suppression or a several hundred-fold enhancement of the responses can be induced (reviewed in refs 14 and 15). IgM enhances responses to large antigens such as erythrocytes, malaria parasites, and keyhole limpet hemocyanine (KLH)^16–19^, but IgM which cannot activate complement loses its enhancing ability^19–21^. Moreover, IgM cannot enhance responses in Cr2 KO mice and optimal enhancement requires expression of CR1/2 both on B cells and FDC^22, 23^.

IgG3 is the most recently discovered feedback-regulator. Passively administered IgG3 enhances antibody responses to small proteins such as ovalbumin (OVA) or bovine serum albumin (BSA)^24–26^. This ability is impaired in Cr2 KO mice^24, 26^ and in mice partially depleted of C3 by treatment with cobra venom factor^24^, but is unperturbed in mice selectively lacking FcγRI^25^, identified as the IgG3-binding Fc-receptor^27^, and in mice lacking all activating FcγRs owing to lack of the common FcRγ chain^24^. Passive administration of specific IgG3 enhances localization of antigen to splenic B cell follicles and binding of antigen to MZ B cells^26^. When MZ B cells are dislocated from the MZ by treatment with FTY720, an antagonist to the sphingosine 1-phosphate receptor S1P1, localization of antigen in the follicles is disrupted. This suggests that transport of IgG3-antigen complexes by MZ B cells explains IgG3-induced localization of antigen to follicles. IgG3 is unable to increase the localization of antigen to follicles in Cr2 KO mice, and optimal enhancement of antibody responses requires that CR1/2 are expressed on both B cells and FDC^26^. Our previous report indicated that IgG3-antigen co-localized with FDC^26^.

We have here further investigated IgG3-mediated deposition of antigen onto FDC and the role of complement factors C1q and C3 in this process. Moreover, the ability of IgG3 to enhance antibody responses to the large protein KLH, with a molecular weight of >7,000 kDa^28^, and to induce immunological memory has been analyzed.

## Results

### Binding of IgG3 immune complexes to MZ B cells is impaired in mice lacking C1q or C3

**Figure 1:**
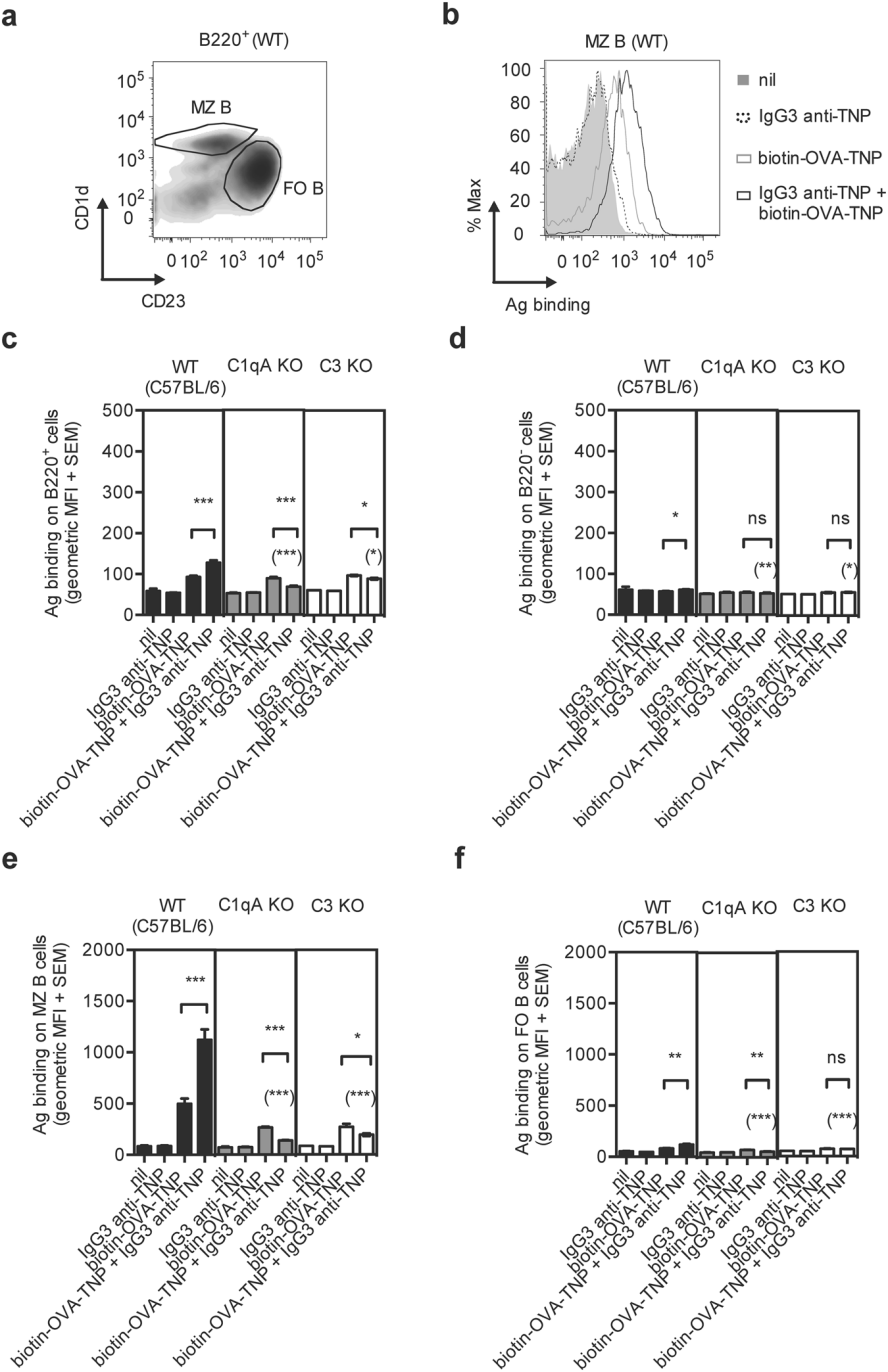
IgG3 increases binding of antigen to MZ B cells in WT, but not in C1qA or C3 KO mice. WT (C57BL/6), C1qA KO, and C3 KO mice were immunized with 50 μg IgG3 anti-TNP (clone IM-F10) alone (n = 1–2), 150 μg biotin-OVA-TNP (n = 5), a mixture of 50 μg IgG3 anti-TNP and 150 μg biotin-OVA-TNP (n = 5), or left unimmunized (nil, n = 1–2). Spleens were harvested 2 h after immunization and single cell suspensions were prepared from half of each spleen. Cells were stained with anti-mouse B220-Pacific Blue, anti-mouse CD23-PE, anti-mouse CD1d-FITC, and SA-APC. (**a**) Representative density plot of WT B220^+^ cells. MZ B cells were gated as B220^+^ CD23^lo^ CD1d^hi^ and FO B cells as B220^+^ CD23^hi^ CD1d^lo^. (**b**) Representative histograms showing binding of antigen to MZ B cells in WT mice immunized with IgG3 anti-TNP + biotin-OVA-TNP (black line), IgG3 anti-TNP (dotted line), biotin-OVA-TNP (grey line), or left unimmunized (nil, grey area). The geometric means of fluorescence intensity (MFI) + SEM of antigen bound to (**c**) total B220^+^ cells, (**d**) B220^−^ cells, (**e**) MZ B cells, or (**f**) FO B cells in WT, C1qA KO, and C3 KO mice are shown. Data are representative of three independent experiments (two with the IgG3 clone IM-F10 and one with IM-H11). The *p* values for comparisons of the responses in mice immunized with antigen alone or IgG3-antigen complexes are indicated without parentheses. The *p* values for comparisons of responses in WT versus C1qA or C3 KO mice immunized with IgG3-antigen complexes are indicated within parentheses. ns, *p* > 0.05; *p < 0.05; **p < 0.01; ***p < 0.001.

Binding of IgG3 immune complexes to MZ B cells is impaired in mice lacking CR1/2^26^. Here we sought to investigate whether classical pathway activation was sufficient to generate the C3 split products required for binding of IgG3-antigen to different B cell populations. Biotin-OVA-2,4,6-trinitrophenyl (TNP), with or without monoclonal IgG3 anti-TNP, was administered to C57BL/6 (wild-type, WT), C1qA KO, and C3 KO mice. Spleens were harvested 2 h later and analyzed by flow cytometry (Fig. 1). IgG3 enhanced binding of antigen to B220^+^ cells (Fig. 1c) and to some extent also to B220^−^ cells (Fig. 1d) from WT, but not from either KO strain. The majority of antigen was found on MZ B cells from WT mice immunized with IgG3-antigen (Fig. 1e). IgG3 increased the binding also to follicular (FO) B cells (Fig. 1f), but the level of antigen was much lower than on MZ B cells. IgG3 did not enhance binding of antigen to MZ or FO B cells in the absence of C1q or C3 (Fig. 1e,f).

Thus, administration of IgG3 caused a preferential binding of antigen to MZ B cells provided either C1q and C3 was present. This suggests that classical pathway activation by IgG3-antigen complexes is sufficient to generate the C3 split products required for their capture by CR1/2^+^ MZ B cells.

### IgG3-mediated antigen localization and deposition on FDC in splenic follicles is impaired in mice lacking C1q or C3

**Figure 2:**
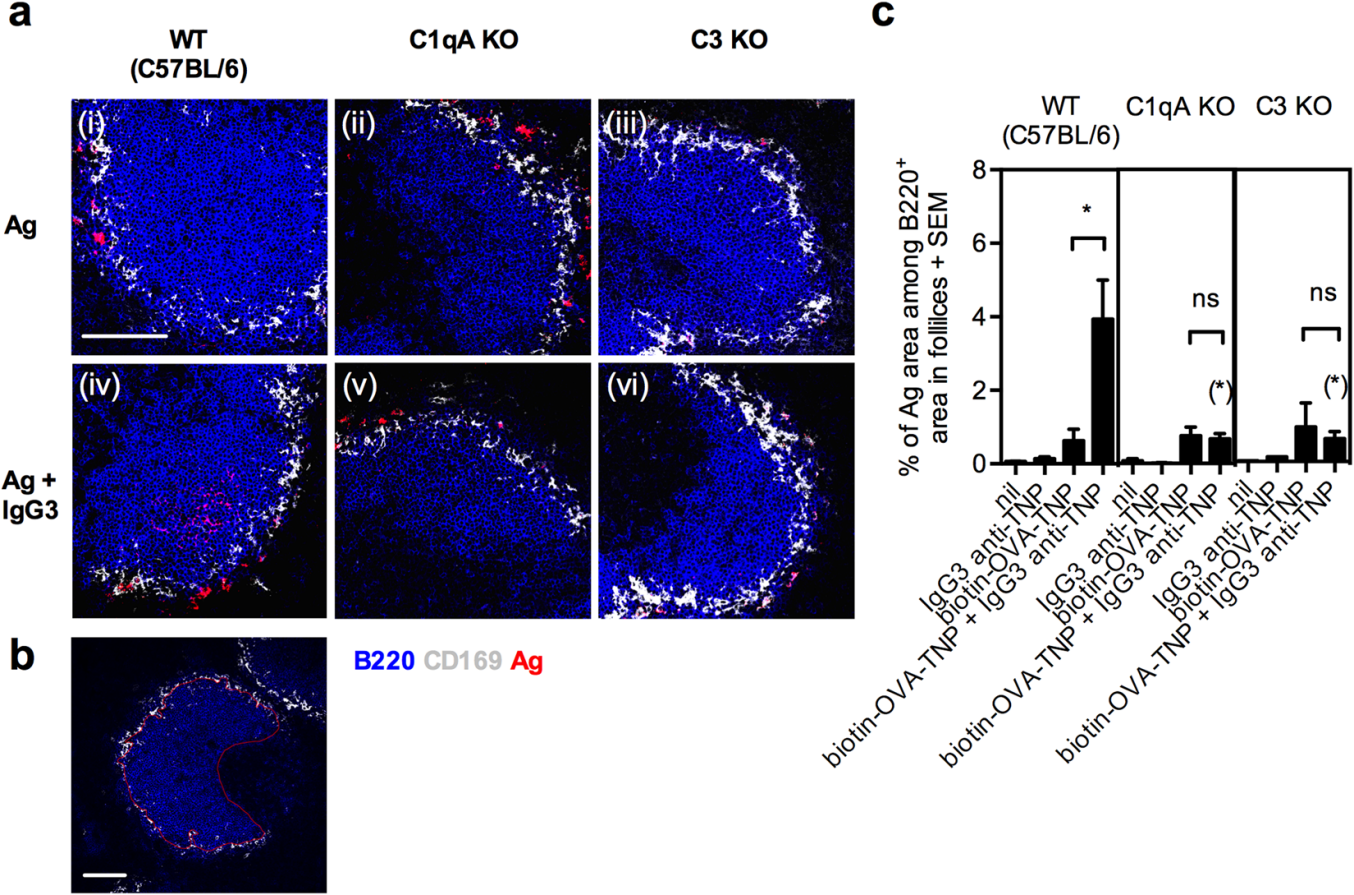
IgG3-mediated localization of antigen to splenic follicles is dependent on C1q and C3. WT (C57BL/6), C1qA KO, and C3 KO mice were immunized as described in the legend to Fig. 1. The other half of each spleen analyzed in flow cytometry (Fig. 1) was sectioned and stained with a mixture of anti-mouse B220-Pacific Blue (B cells; blue), anti-mouse CD169-FITC (metallophilic macrophages; grey), and SA-APC (antigen; red) for 1 h. One longitudinal section of each half spleen was analyzed by confocal microscopy. (**a**) Representative FO areas (313 μm × 313 μm, scale bar = 100 μm) of 4–5 follicles per mouse from each group. (**b**) The red line shows how the border of the follicle against the MZ and the T zone was defined (640 μm × 640 μm, scale bar = 100 μm). (**c**) Quantification of antigen concentration in the B cell follicles with borders as defined in (**b**). Data are representative of three independent experiments (two with IgG3 clone IM-F10 and one with IM-H11) and are shown as mean + SEM. The *p* values for comparisons of the responses in mice immunized with antigen alone or IgG3-antigen complexes are indicated without parentheses. The *p* values for comparisons of responses in WT versus C1qA or C3 KO mice immunized with IgG3-antigen complexes are indicated within parentheses. ns, *p* > 0.05; *p < 0.05.

The other halves of the same spleens that were analyzed by flow cytometry in Fig. 1, were subjected to confocal microscopy to determine the amount of antigen present in B cell follicles (Fig. 2). Immunization with antigen alone resulted in very low levels of antigen in the follicles, regardless of the mouse strain. In contrast, substantial amounts of antigen were seen inside the follicles in WT mice immunized with IgG3-antigen. In complement-deficient mice, IgG3-antigen did not enter follicles but remained in the MZ.

**Figure 3:**
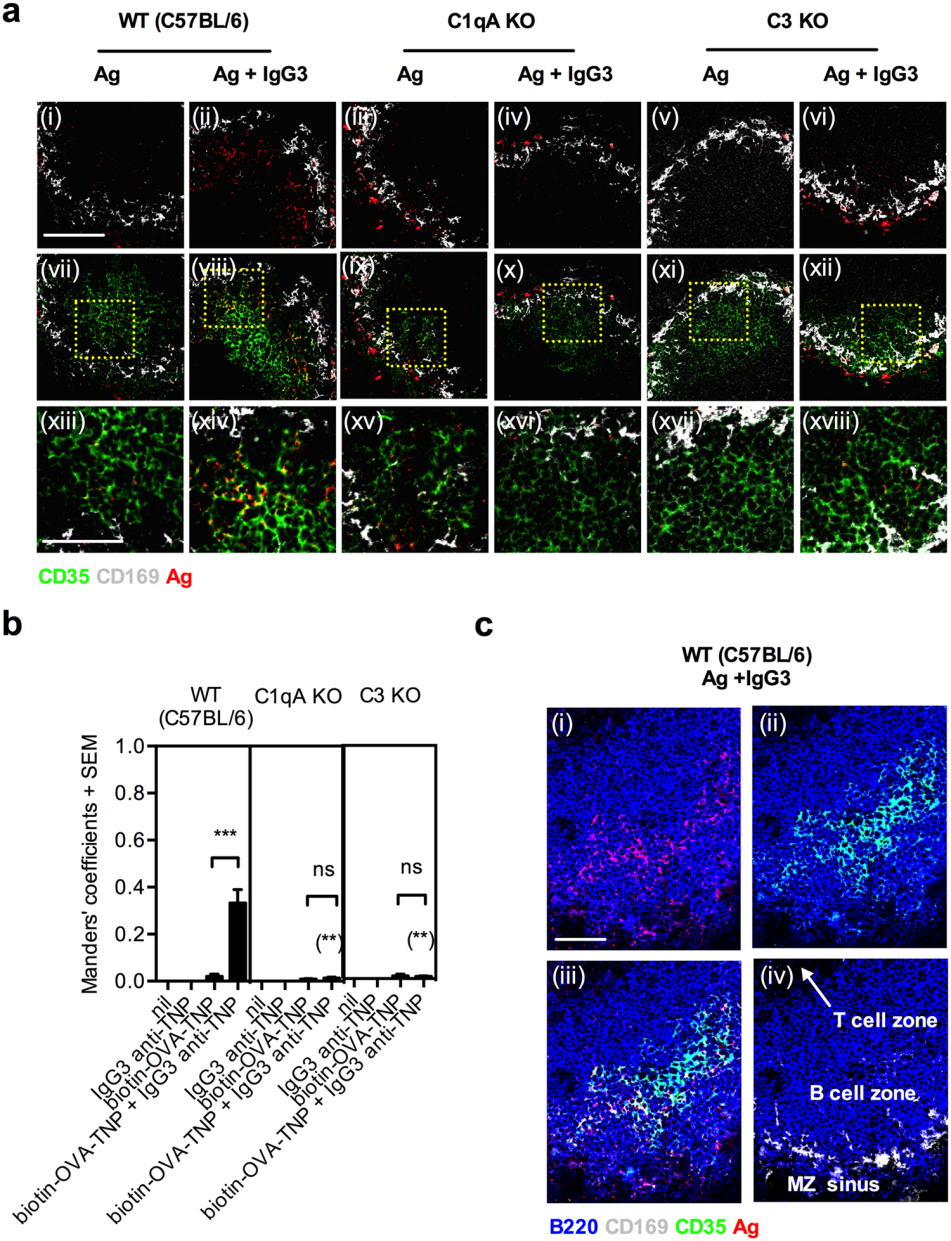
IgG3-mediated antigen deposition on FDC is impaired in mice lacking C1q or C3. Spleen sections from WT (C57BL/6), C1qA KO, and C3 KO mice described in Fig. 2 were here stained with rat anti-mouse CD35/CR1 for 1 h, followed by PE-labeled goat anti-rat IgG (FDC; green) for 1 h, blocked with 5% rat serum for 30 min, and then stained with a mixture of anti-mouse B220-Pacific blue (B cells; blue), anti-mouse CD169-FITC (metallophilic macrophages; grey) and SA-APC (antigen; red) for 1 h. One longitudinal section from each half spleen was analyzed by confocal microscopy. (**a**) (i–xii) Images show representative FO areas (250 μm × 250 μm, scale bar = 100 μm) of 4–5 follicles per mouse. (i–vi) Antigen and metallophilic macrophages are shown. (vii–xii) Antigen, metallophilic macrophages, and FDC are shown. (xiii–xviii) Magnifications (94 μm × 94 μm, scale bar = 50 μm) of the fields indicated by dotted squares in vii–xii. (**b**) Quantification of the fraction of total FDC area staining positive also for antigen (antigen^+^ FDC area/total FDC area) is shown as Manders’ coefficients + SEM. (**c**) Representative images (200 μm × 250 μm, scale bar = 50 μm) of a follicle from a C57BL/6 mouse immunized with IgG3 anti-TNP and biotin-OVA-TNP. (i) B cells and antigen are shown. (ii) B cells and FDC are shown. (iii) B cells, FDC and antigen are shown. (iv) Outline of a follicle indicated by B cells and metallophilic macrophages (anti-CD169 staining). Data are representative of three independent experiments (two with clone IM-F10 and one with clone IM-H11) and are shown as mean + SEM. The *p* values for comparisons of the responses in mice immunized with antigen alone or with IgG3-antigen complexes are indicated without parentheses. The *p* values for comparisons of responses in WT versus C1qA or C3 KO mice immunized with IgG3-antigen complexes are indicated within parentheses. ns, *p* > 0.05; *p < 0.05; **p < 0.01; ***p < 0.001.

Previous data suggested that IgG3-antigen complexes were deposited onto FDC in the B cell follicle, but whether or not this step involved complement was not investigated^26^. When spleen sections were stained with a monoclonal antibody recognizing CR1, which is highly expressed on FDC^3^, the typical FDC network (green) was prominent in all mice (Fig. 3a). The antigen (red) frequently co-localized with FDC of WT but not complement-deficient mice immunized with IgG3-antigen (yellow areas in Fig. 3a(viii) and (xiv)). Quantification demonstrated that antigen was detected on 33% of the total FDC area (Manders’ coefficients) in WT mice (Fig. 3b). In mice immunized with IgG3-antigen complexes, there was a strikingly similar pattern of the areas staining positive for FDC and for antigen (Fig. 3c). Calculating the ratio between the areas of antigen localized on FDC (light) and the total antigen area (red) within follicles, Manders’ coefficients of 0.5–0.9 were obtained. These were based on analysis of 4–5 follicles per mouse in 3 independent experiments with 5 mice per experiment. The overlap between the antigen and FDC areas is shown on a tile scan image of a larger section of a spleen in Supplementary Fig. 1.

In summary, IgG3-complexed antigen can be found in follicles of WT but not complement-deficient mice. Two hours after immunization, approximately one third of the total FDC area is occupied by antigen and 50–90% of the antigen that reach the follicles are found on FDC.

### IgG3-mediated enhancement of antibody responses is impaired in mice lacking C1q or C3

**Figure 4:**
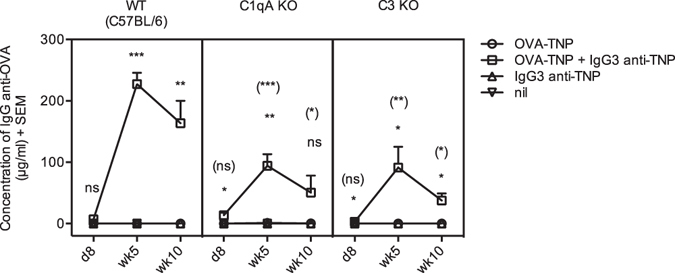
IgG3-mediated enhancement of antibody responses is impaired in mice lacking C1q or C3. WT (C57BL/6), C1qA KO, and C3 KO mice were immunized with 50 μg IgG3 anti-TNP (IM-F10) alone (n = 1–2), 20 μg OVA-TNP (n = 5), a mixture of 50 μg IgG3 anti-TNP (IM-F10) and 20 μg OVA-TNP (n = 5), or left untreated (nil; n = 1–2). Mice were bled at indicated time points. Sera were diluted 1:25 (in antigen, IgG3 alone, and nil groups), or 1:3125 (in IgG3 + antigen-group), and screened for OVA-specific IgG in ELISA. Comparisons between WT and C1qA KO mice were done in 3 experiments and between WT and C3 KO mice in 2 experiments and a representative experiment is shown. The *p* values for comparisons of the responses in mice immunized with antigen alone or IgG3-antigen complexes are indicated without parentheses. The *p* values for comparisons of responses in WT versus C1qA or C3 KO mice immunized with IgG3-antigen complexes are indicated within parentheses. ns, *p* > 0.05; *p < 0.05; **p < 0.01; ***p < 0.001.

No reduction in antibody responses to IgG3-antigen complexes was observed in FcγR-deficient mice, demonstrating that the effect is independent of these receptors^24, 25^. However, the antibody response to IgG3-complexed antigens is markedly reduced, although not completely absent, in mice lacking CR1/2^24, 26^. To analyze whether involvement of other receptors for the subfragments of C3 than CR1/2 could explain the residual enhancement seen in Cr2 KO mice, IgG3-mediated enhancement in C3 KO mice was determined. C1qA KO mice were also included to elucidate the involvement of classical pathway activation. As expected, IgG3 administered together with OVA-TNP enhanced the antibody production in WT mice (Fig. 4). The enhancement by IgG3 was significantly reduced, but not completely abrogated, in C3 and C1qA KO mice (Fig. 4). These data confirm that complement plays an important role in IgG3-mediated enhancement, but also show that a reduced enhancement can take place in the absence of C1q or C3.

### IgG3 enhances induction of immunological memory

**Figure 5:**
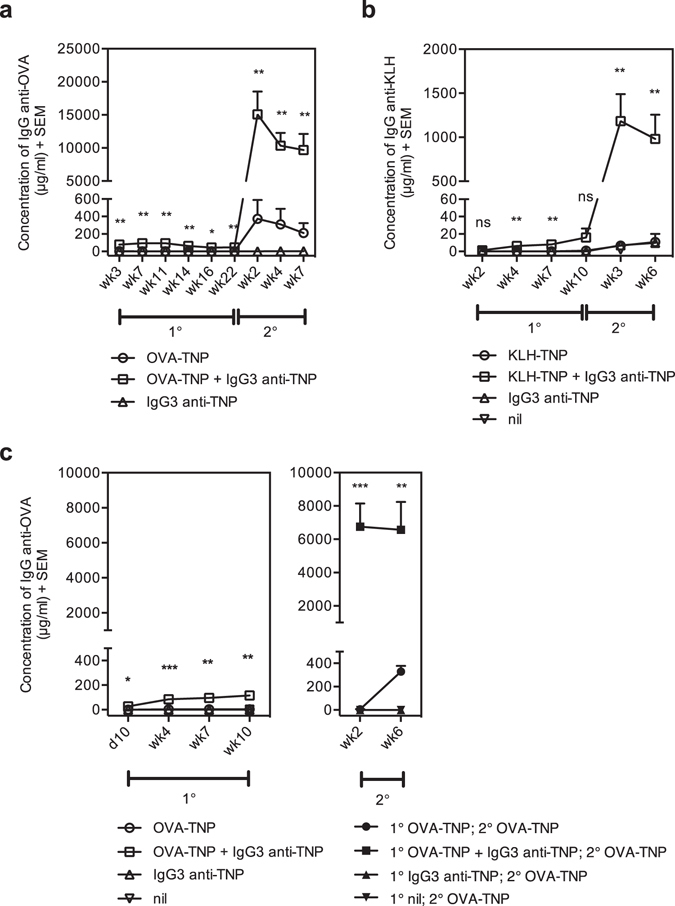
IgG3 enhances induction of immunological memory. (**a**) BALB/c mice were primed *i.v*. with 50 μg IgG3 anti-TNP (IM-F10) alone (n = 3), 20 μg OVA-TNP (n = 6), or a mixture of 50 μg IgG3 anti-TNP (IM-F10) and 20 μg OVA-TNP (n = 6). Twenty-two weeks after priming, all mice were boosted *i.v*. with 20 μg OVA-TNP. (**b**) BALB/c mice were primed *i.v*. with 50 μg IgG3 anti-TNP (IM-F10) alone (n = 3), 10 μg KLH-TNP (n = 6), a mixture of 50 μg IgG3 anti-TNP (IM-F10) and 10 μg KLH-TNP (n = 6), or left untreated (nil, n = 1). Ten weeks after priming, all mice were boosted *i.v*. with 10 μg KLH-TNP. (**c**) BALB/c mice were primed *i.v*. with 50 μg IgG3 anti-TNP (IM-F10) alone (n = 2), 20 μg OVA-TNP (n = 3), or a mixture of 50 μg IgG3 anti-TNP (IM-F10) and 20 μg OVA-TNP (n = 3), or left unimmunized (nil, n = 1). Ten weeks after priming, single cell suspensions of the spleens from each group were prepared and 15 × 10^6^ cells/mouse were transferred to naïve BALB/c mice that had been whole-body irradiated the day before. All recipients were “boosted” *i.v*. with 20 μg OVA-TNP immediately after transfer. (**a–c**) Mice were bled at the indicated time points. Sera were diluted 1:25 (in all antigen alone and IgG3 alone groups), 1:625 (in IgG3 + antigen-group in the primary-response phase) or 1:78125 (in IgG3 + antigen-group in the secondary-response phase) and screened in ELISA. (**a**) is representative of three experiments (two in BALB/c and one in C57BL/6 mice). (**b**) and (**c**) are representative of two experiments each in BALB/c mice. The *p* values for comparisons of the responses in mice immunized with antigen alone or IgG3-antigen complexes are indicated. ns, *p* > 0.05; *p < 0.05; **p < 0.01; ***p < 0.001.

IgG3 enhances primary antibody responses against small soluble proteins (OVA and BSA)^24–26^, but whether IgG3 can enhance induction of memory responses or responses to large proteins has not been investigated. To this end, BALB/c mice were primed with OVA-TNP alone or together with TNP-specific IgG3, and boosted with OVA-TNP alone after 22 weeks. Priming with IgG3 together with antigen led to a significantly higher primary as well as secondary antibody response as compared to priming with antigen alone (Fig. 5a). Similar experiments, using the large protein antigen KLH-TNP, demonstrated that IgG3 could enhance primary responses as well as priming for a secondary response against KLH (Fig. 5b). Not only passively administered, but also endogenously produced antibodies can feedback regulate immune responses^29, 30^. Therefore, the enhanced secondary responses observed after *in situ* booster immunizations (Fig. 5a and b) might be due to endogenous feedback regulation mediated by the high concentrations of antigen-specific IgG generated in the IgG3-antigen-primed mice. To verify that memory cells had indeed been induced after priming, boosting was also performed in an adoptive transfer system (Fig. 5c). Mice were primed with either OVA-TNP alone or with IgG3 anti-TNP + OVA-TNP. After 22 weeks, splenocytes were harvested and transferred to irradiated naïve mice and all recipients were boosted with OVA-TNP alone. The secondary antibody responses in recipients of splenocytes from IgG3 anti-TNP + OVA-TNP-primed mice were significantly higher than responses in recipients of splenocytes from OVA-TNP-primed mice (Fig. 5c), demonstrating that priming with IgG3 together with antigen enhances the development of memory cells. In conclusion, IgG3 is able to enhance priming for immunological memory against OVA as well as KLH.

## Discussion

We here demonstrate that IgG3-antigen complexes, administered to WT but not to C1qA KO or C3 KO mice, bound to MZ B cells and were found in follicles two hours after administration. Antigen administered alone bound less efficiently to MZ B cells and was not found in the follicles (Figs 1 and 2). The crucial role of C1q shows that classical pathway activation is required. The distribution pattern of antigens in the IgG3-antigen-immunized mice is strikingly similar to that of FDC (Fig. 3c) and between 50% and 90% of the antigen detected in follicles co-localized with FDC, covering 33% of the FDC area. Together with our previous finding that dislocation of MZ B cells prevents IgG3-mediated localization of antigen in the follicles^26^, this suggests that these cells capture IgG3-antigen-complement complexes and deposit them onto FDC during their shuttling to and from the follicles^7, 8^. The scenario strongly resembles what is seen after administration of IgM-antigen complexes, which are also transported into follicles by MZ B cells and are subsequently deposited onto FDC provided C3 and CR1/2 are present^6, 19^. An interesting question is whether competition between various immune complexes, transported into the follicles, will affect the composition and amount of antigen bound to an FDC and influence the resulting antibody response. Should this be the case, it could partly explain the old phenomenon of antigenic competition^31, 32^. Epitope masking by IgM antibodies has been suggested to play an important role in B cell selection in germinal centers^33^ and can also modulate antibody responses to viruses^34^, but whether IgG3 has these effects is not known.

Monoclonal antigen-specific IgE antibodies, passively administered together with OVA or BSA, can also feedback-enhance antibody responses. This effect requires the presence of the low affinity receptor for IgE, CD23. Available data suggest that IgE-antigen complexes are captured by recirculating CD23^+^ FO B cells, which transport the antigen to splenic B cell follicles. In the spleen, “IgE-delivered” antigen is taken up by CD8α^−^ conventional dendritic cells via unknown mechanisms and subsequently presented to CD4^+^ T cells^35–38^. The current observation that the antigen within the follicles of WT mice immunized with IgG3-OVA-TNP immune complexes was almost exclusively found in the FDC areas differs from what was observed in follicles after immunization with IgE-OVA/OVA-TNP immune complexes. In these mice, antigen localized diffusely over the entire follicles and was not preferentially found in the FDC areas^36, 38^. The lack of obvious involvement of FDC in this situation agrees well with the finding that IgE-mediated enhancement of antibody responses requires expression of this receptor on B cells, but not on FDC^35, 39^. In contrast, IgM- and IgG3-mediated enhancement requires expression of CR1/2 on both B cells and FDC for optimal effect^23, 26^. IgE-antigen complexes are transported into follicles by recirculating CD23^hi^CD21^lo^ FO B cells^36^, while IgG3- and IgM-antigen complexes are transported by CD23^low^CD21^hi^ MZ B cells^6, 26^. Hypothetically, MZ B cells may be programmed to migrate towards FDC while FO B cells are not, explaining why the latter deposit their antigen cargo randomly in the follicles.

Not only IgG3, but also the other IgG subclasses, are able to feedback enhance antibody responses against soluble proteins. The enhancing effect of IgG1, IgG2a, and IgG2b is dependent on activating FcγRs^40^ but independent of complement and CR^22, 41^. An interesting question is why different IgG isotypes, the majority of which are able to both activate complement and bind to FcγRs, preferentially use only one of these effector functions in their feedback enhancement of antibody responses. IgG3 may not use FcγRs because it only binds to one of the activating FcγRs, FcγRI, and because its affinity for the receptor is rather low (5-fold lower than that of IgG2a)^27^. The majority of IgG1 antibodies does not activate complement and therefore is expected to operate via Fc-receptors. The reason why IgG2a and IgG2b preferentially use FcγRs may depend on the difficulty for these isotypes to activate the classical pathway *in vivo*. For reasons of avidity, at least two IgG molecules are required to fix C1q. Unless very high concentrations of e.g. specific IgG2a are present, the probability that two IgG2a molecules bind close enough on the same antigen to capture a C1q molecule may be quite low and may not occur until late in an immune response. On the other hand, one single IgG3 molecule binding to a surface will attract other IgG3 molecules through its capacity of cooperative binding via Fc-Fc interactions^42–44^, thereby increasing the chances for IgG3 to capture C1q early in an immune response. For these reasons, we do not find it surprising that IgG3 preferentially uses complement while the other IgG subclasses rely on FcγRs.

When Cr2 KO mice^24, 26^ or mice depleted of C3 by treatment with cobra venom factor^24^ were immunized with IgG3-antigen, the antibody responses were much lower than in WT mice. However, a minor enhancement remained. As involvement of FcγRs has been excluded^24, 25^, we sought to investigate whether other complement receptors than CR1/2 could explain the phenomenon. In C3 KO mice, all three complement-activation pathways are blocked, and therefore generation of ligands to all receptors for complement split products downstream of C3 should be abrogated. However, although C3 KO mice exhibited a reduced antibody response after immunization with IgG3-antigen, a minor enhancement still remained in these animals (Fig. 4), thus resembling the findings in Cr2 KO mice^26^. This observation excludes that receptors for complement split products downstream of C3 are involved in the residual enhancement seen in Cr2 KO, C1qA KO, and C3 KO mice. We have no definite explanation for this finding but, as IgG3 easily aggregates^45^, it may be due to formation of IgG3-antigen aggregates which are more immunogenic than single OVA-TNP molecules.

It is here for the first time demonstrated that priming with IgG3-OVA-TNP or IgG3-KLH-TNP complexes results in a much stronger secondary antibody response after re-stimulation with antigen than does priming with antigen alone (Fig. 5). This would be an important function in a physiological situation. It seems feasible that IgG3 and IgG1/2a/2b complement each other during induction of antibody responses by using complement or FcγRs respectively. IgG3 may preferentially act early and the other subclasses late during the response. IgM and IgG3 both enhance antibody responses via the complement system, but may complement each other regarding which types of antigen they prefer: IgM enhances antibody responses to large antigens such as erythrocytes, malaria parasites, and KLH, while IgG3 enhances responses to small proteins like OVA and BSA, and, as shown herein, also to KLH. IgM’s preference for large antigens could hypothetically be explained by the fact that it relies on a conformational change to be able to bind C1q^46^, and this may require binding to an antigen larger than the IgM molecule itself.

IgG3 is an IgG subclass with many intriguing features. It is a cryoglobulin with a strong tendency to self-aggregate^42, 43, 47^. It is the predominant IgG subclass in responses to thymus-independent type 2 antigens^48, 49^. Mice lacking IgG3 are more susceptible to pneumococcal infections than WT mice^50^, but it has not been shown whether natural IgG3 feedback enhances antibody responses during infections. Natural IgM on the other hand plays a role in defenses against bacterial infections^51^ as well as in antibody production^52, 53^. IgG3 is also associated with autoimmune diseases, and e.g. in a mouse model for systemic lupus erythematosus, mice lacking IgG3 have an attenuated disease progress^54^. Our demonstration that IgG3 via complement induces a rapid and efficient deposition of antigen on FDC and enhances primary and secondary antibody responses to a wide range of proteins may aid in understanding how IgG3 exerts its effector functions during infections and autoimmune conditions.

## Materials and Methods

### Mice

C57BL/6JBomTac (C57BL/6) mice were obtained from Taconic Bioscience, Inc. (Hudson, NY, USA). C1qA KO mice (lacking the entire C1q molecule) on C57BL/6 background were obtained from Dr. Marina Botto (Imperial College London, United Kingdom)^55^. C3 KO mice on C57BL/6 background were obtained from Jackson Laboratories (Bar Harbor, ME, USA). BALB/c mice were from Bommice (Ry, Denmark). Cr2 KO mice (lacking CR1/2) on C57BL/6 background were a gift from Dr. Hector Molina^56^ and were backcrossed for 10 generations to BALB/c mice^22^. All mice were bred and maintained at the National Veterinary Institute (Uppsala, Sweden) under the supervision of a veterinarian. All animal experiments were performed with the approval of Uppsala Animal Research Ethics Committee and all methods were performed in accordance with the relevant guidelines and regulations. Mice were at least six weeks old at the onset of the experiments and were age and sex matched within each experiment.

### Antigens

OVA and TNP were obtained from Sigma-Aldrich (St Louis, MO, USA). Conjugation of OVA to TNP was performed as described^57^ and conjugates with 1.9 TNP molecules/OVA molecule were used throughout. Biotinylation of OVA-TNP (biotin-OVA-TNP) was performed as described^36^ with slight modifications^26^. KLH-TNP (with 23 TNP molecules/KLH molecule) was purchased from Biosearch Technologies Inc. (Petaluma, CA, USA).

### Antibodies used in flow cytometry and confocal microscopy

In flow cytometry analysis, rat IgG2bκ anti-mouse CD16/CD32 (clone 2.4G2; BD Pharmingen, San Diego, CA, USA) was used to block the Fc receptors. Pacific Blue-labeled rat IgG2aκ anti-mouse CD45R (B220) (clone RA3–6B2; BD Pharmingen), FITC-labeled rat IgG2aκ anti-mouse CD23 (clone B3B4; eBioscience), PE-labeled rat (LEW) IgG2bκ anti-mouse CD1d (CD1.1, Ly-38) (clone 1B1; BD Pharmingen) and streptavidin (SA)- allophycocyanin (APC) (eBioscience) were used to detect antigen bound to B cells in the spleen samples. In confocal microscopy analysis, Pacific Blue-labeled rat IgG2aκ anti-mouse CD45R (B220) (clone RA3-6B2; BD Pharmingen), FITC-labeled rat IgG2a anti-mouse CD169 (clone MOMA-1; AbD Serotec, Oxford, UK), and SA-APC (eBioscience) were used to detect antigen localization in the follicles. Purified rat IgG2a anti-mouse CD35/CR1 (clone: 8C12^58^, in house isolated and purified as decribed)^26^ and PE-labeled goat anti-rat IgG (BD Pharmingen) were included to detect FDC.

### Immunization and blood sampling

Murine monoclonal IgG3 anti-TNP (clones IM-F10 and IM-H11) were isolated from B cell hybridoma supernatants and stored as described^24, 25^. Indicated amounts of OVA-TNP, biotin-OVA-TNP, or KLH-TNP were pre-mixed with IgG3 anti-TNP for 1 h at room temperature before intravenous administration in tail veins (in a total volume of 200 µl per mouse). Blood samples were collected from the tail arteries.

### Flow cytometry

Splenocytes were prepared as described^21^. In short, single cell suspensions from spleen samples were depleted of RBC, washed in PBS, re-suspended in FACS buffer (PBS with 2% fetal bovine serum, Sigma-Aldrich), and incubated with anti-Fc receptor antibodies before staining. Samples were stained with pre-mixed antibodies (as indicated in the legend to Fig. 1) and fixed with 4% paraformaldehyde in PBS before analysis. Lymphocytes were gated according to their forward- and side scatter properties and at least 10^5^ events (lymphocytes) per sample were recorded on a Fortessa cytometer (BD Biosciences) at the Biovis platform (SciLifeLab, Uppsala University). Data were analyzed using the FlowJo software (Tree Star Inc., Ashland, OR, USA).

### Confocal microscopy

Spleen samples were prepared, sectioned and stored as described^21^. Briefly, they were embedded in optimum cutting temperature (O.C.T) compound (VWR International, Radnor, PA, USA), snap frozen in liquid nitrogen, and stored at −80 °C. Samples were cut into 8 μm sections using a CryoStar NX70 Cryostat (Thermo Fisher Scientific, Waltham, MA, USA), thaw-mounted on Thermo Scientific Menzel-Gläser Microscope Slides (Thermo Fisher Scientific), air-dried and stored at −80 °C. Before staining, slides were fixed 30 s in 50% acetone (Sigma-Aldrich), followed by 5 min in 100% acetone (Sigma-Aldrich) and finally rehydrated in PBS for 15 min. Slides were stained (as indicated in the legends of Figs 2 and 3, and Supplementary Fig. 1) at room temperature in the dark and washed twice in PBS in between each staining step. Finally, slides were mounted with Fluoromount G (Southern Biotech, Birminghan, AL, USA) and stored at 4 °C. Slides were analyzed using a LSM 700 confocal microscope (Carl Zeiss, Thornwood, NY, USA) and images were processed by software ImageJ (NIH, Bethesda, MD, USA).

### Enzyme-linked immunosorbent assay (ELISA)

Determination of OVA- or KLH-specific IgG in sera was performed by ELISA as described^35^. Costar 96-well EIA/RIA (Sigma-Aldrich) or high-binding ELISA plates (Sarstedt, Hildesheim, Lower Saxony, Germany) were pre-coated with OVA or KLH overnight at 4 °C. After washing, appropriately diluted sera were added to the plates and incubated overnight at 4 °C. After washing, phosphatase-conjugated sheep anti-mouse IgG (diluted 1:1000; Jackson ImmunoResearch Laboratories, West Grove, PA) was added and the plates incubated at room temperature for 3 h. After washing, phosphatase substrate (Sigma-Aldrich) diluted in diethanolamine buffer was added to the plates. The absorbance at 405 nm was measured after 30–120 min of incubation at room temperature in the dark. Data were analyzed using the SOFTmax software (Molecular Devices, Sunnyvale, CA, USA) and results were presented as μg/ml after calculations based on a standard curve. For OVA-specific ELISA (Figs 4 and 5), a polyclonal IgG anti-OVA standard serum with a starting concentration of 0.295 μg/ml was used. For KLH- specific ELISA (Fig. 5), a polyclonal IgG anti-KLH standard serum with a starting concentration of 0.02 mg/ml was used.

### Spleen cell transfer

Female naïve BALB/c mice were whole-body irradiated with a sub-lethal dose of 7.5 Gy and rested for 24 hours before *i.v*. transfer of 15 × 10^6^ spleen cells from female BALB/c mice, primed 10 weeks before as described in the legend to Fig. 5. All recipients were boosted *i.v*. with 20 μg OVA-TNP in 200 μl PBS immediately after transfer.

### Statistical analysis

Statistical differences between groups were determined by unpaired Student’s *t*-test (two tailed): ns (not significant), *p* > 0.05; *p < 0.05; **p < 0.01; ***p < 0.001. The co-localization analyses were shown as Manders’ coefficients, which represented the fraction of co-localized signals in indicated channel of dual-color confocal images^59^ (Fig. 3).

## Electronic supplementary material

Supplementary Information
