# Supplementary material for: IgG3-antigen complexes are deposited on follicular dendritic cells in the presence of C1q and C3
Source: Sci Rep. 2017 Jul 14;7:5400. doi: 10.1038/s41598-017-05704-3 (PMC5511153; doi:10.1038/s41598-017-05704-3)
Supplement: Supplementary file 1 — Supplementary Information [file 41598_2017_5704_MOESM1_ESM.pdf]

IgG3-antigen complexes are deposited on follicular dendritic cells in the presence of C1q and C3

Lu Zhang[1], Zhoujie Ding[1], and Birgitta Heyman[1,*]

[1] Department of Medical Biochemistry and Microbiology, Uppsala University, Box 582, BMC, SE-751 23 Uppsala, Sweden

[*] Corresponding author

Supplementary Figure 1. The vast majority of intra-follicular antigen is co-localized with FDC. Spleen sections from WT (C57BL/6) mice immunized with (i-iv) 50 μg IgG3 anti-TNP (clone IM-H11) + 150 μg biotin-OVA-TNP or with (v-viii) 150 μg biotin-OVA-TNP alone were stained as described in Fig. 3. One longitudinal section from each half spleen was analyzed by confocal microscopy. Representative images (2483 μm × 1625 μm, scale bar = 500 μm) from each group are shown. (i, v) Anti-B220 staining (B cells) and anti-CD169 staining (metallophilic macrophages) are shown. (ii, vi) Anti-CR1 staining (FDC) and metallophilic macrophages are shown. (iii, vii) Antigen and metallophilic macrophages are shown. (iv, viii) Antigen, FDC, and metallophilic macrophages are shown.

Substantial amount of antigen is seen in follicles of mice immunized with IgG3 + antigen (iii) but not with antigen alone (vii). Notably, the majority of intrafollicular antigen is found in the FDC area, the overlap between red and green resulting in yellow staining (iv). Antigen that does not co-localize to FDC is almost exclusively found in the MZ outside the border defined by metallophilic macrophages (iv).

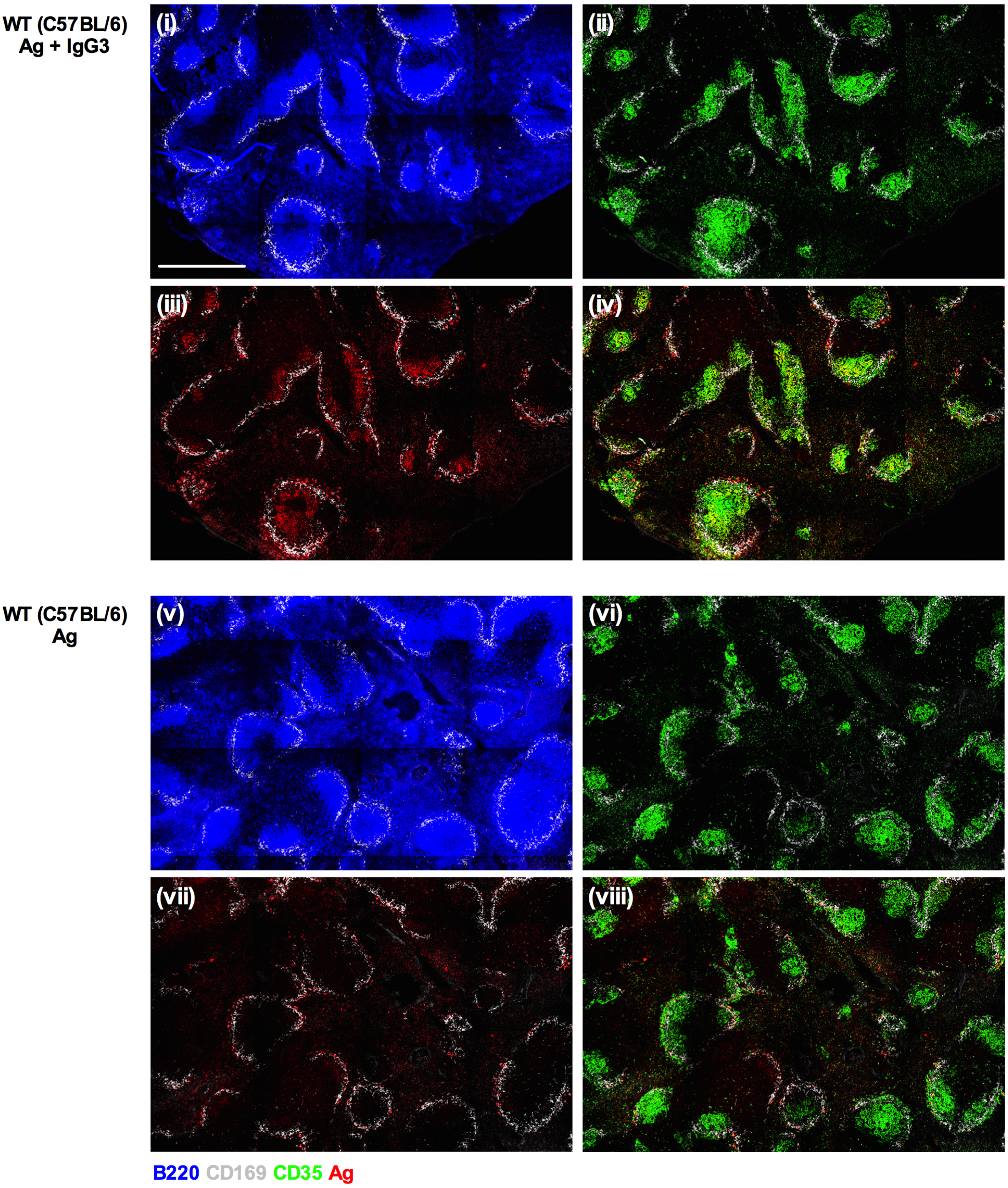
WT (C57BL/6)
Ag + IgG3
(i)
(ii)
(iii)
(iv)
WT (C57BL/6)
Ag
(v)
(vi)
(vii)
(viii)
B220 CD169 CD35 Ag
